# Patient satisfaction with telemedicine in the Philippines during the COVID-19 pandemic: a mixed methods study

**DOI:** 10.1186/s12913-023-09127-x

**Published:** 2023-03-22

**Authors:** Alicia Victoria G. Noceda, Lianne Margot M. Acierto, Morvenn Chaimek C. Bertiz, David Emmanuel H. Dionisio, Chelsea Beatrice L. Laurito, Girrard Alphonse T. Sanchez, Arianna Maever Loreche

**Affiliations:** 1 School of Medicine and Public Health, Ateneo de Manila University, Pasig City, Philippines; 2 National Clinical Trials and Translation Center, National Institutes of Health, University of the Philippines Manila, Manila, Philippines

**Keywords:** COVID-19, Patient satisfaction, Telehealth, Telemedicine, Universal health care, Philippines

## Abstract

**Background:**

The capacity to deliver essential health services has been negatively impacted by the COVID-19 pandemic, particularly due to lockdown restrictions. Telemedicine provides a safe, efficient, and effective alternative that addresses the needs of patients and the health system. However, there remain implementation challenges and barriers to patient adoption in resource-limited settings as in the Philippines. This mixed methods study aimed to describe patient perspectives and experiences with telemedicine services, and explore the factors that influence telemedicine use and satisfaction.

**Methods:**

An online survey consisting of items adapted from the Consumer Assessment of Healthcare Providers and Systems (CAHPS) Clinician & Group Adult Visit Survey 4.0 (beta) and the Telehealth Usability Questionnaire (TUQ) was completed by 200 participants aged 18 to 65 years residing in the Philippines. A subsample of 16 participants was interviewed to provide further insights on their experiences. We used descriptive statistics to analyze survey data and thematically analyzed data from interviews guided by the principles of grounded theory.

**Results:**

Participants were generally satisfied with telemedicine, and found it to be an efficient and convenient means of receiving healthcare. About 3 in 5 perceived telemedicine as affordable, with some finding telemedicine costs to be high and comparable to in-person consultations. Our results suggest that participants preferred telemedicine services, especially in cases where they feel that their condition is not urgent and does not need extensive physical examination. Safety against COVID-19, privacy, accessibility, and availability of multiple communication platforms contributed to patient satisfaction with telemedicine. Negative perceptions of patients on quality of care and service related to their telemedicine provider, inherent limitations of telemedicine in the diagnosis and management of patients, perceived high costs especially for mental health conditions, and poor connectivity and other technological issues were barriers to telemedicine use and satisfaction.

**Conclusion:**

Telemedicine is viewed as a safe, efficient, and affordable alternative to receiving care. Expectations of patients on costs and outcomes need to be managed by providers to increase satisfaction. Continued adoption of telemedicine will require improvements in technology infrastructure and technical support for patients, training and performance evaluation of providers to ensure quality of care and service, better patient communication to meet patient needs, and integration of telemedicine services in remote areas that have limited access to medical services. Telemedicine, to realize its full potential, should be centered in health equity – addressing patient barriers and needs, reducing health disparities across population groups and settings, and providing quality services to all.

**Supplementary Information:**

The online version contains supplementary material available at 10.1186/s12913-023-09127-x.

## Background

Telemedicine involves the use of technology in the diagnosis, treatment, monitoring, and management of patients [1], including video teleconferencing, remote patient monitoring, mobile health applications, and more traditional methods of communication such as text, email, voice and video calls [2]. It is a safe, efficient, and affordable alternative to in-person healthcare services, which benefits both the patient and health system. Patients are able to access high-quality healthcare for non-urgent conditions without visiting a health facility [2–4], thereby saving resources and reducing unnecessary burden on the health system [1]. It has also been shown to be effective in improving health outcomes and lowering risks of hospitalization and readmission [5–7].

The COVID-19 pandemic has negatively impacted the capacity of health systems to deliver essential services, especially among low-and-middle income countries such as the Philippines [8, 9]. As cases increased and lockdown restrictions were imposed in the country, hospital admissions and procedures declined for non-COVID-19 and 12 high-burden diseases [10]. To respond to this public health crisis, the use of telemedicine has rapidly expanded to respond to the needs of different population groups, health conditions, and settings including urban areas [11].

Despite the benefits of telemedicine, challenges related to the patient (e.g., costs, access), health provider and system (e.g., financial and time constraints), and external factors such as poor connectivity pose problems to its wide-scale implementation [6, 8, 12]. Barriers to patient adoption, in particular, need to be addressed to encourage continued use. Studies on factors influencing telemedicine use and satisfaction have provided recommendations on how to improve the design and delivery of quality telemedicine services [13, 14]. To date however, only three local studies have documented patient perspectives and experiences with telemedicine services during the pandemic and all reported good levels of satisfaction [15–17]. Factors negatively influencing telemedicine use and satisfaction were not explored due to the quantitative nature of the studies conducted, and data were only collected from Filipinos residing in Luzon – one of the three main island groups in the country – potentially missing out on experiences from other regions and more rural settings.

With the transition of the Philippine health system to Universal Health Care (UHC) [18, 19], telemedicine adoption will be critical in achieving the UHC goal of providing healthcare to all by overcoming geographical barriers and delivering care to even the most disadvantaged communities [20]. Our study builds on existing evidence by using a mixed methods approach to give further context on factors influencing patient adoption of telemedicine throughout the country. By better understanding patient experience with telemedicine including barriers and satisfaction, this study may provide insights into opportunities for integrating telemedicine into routine care and improving telemedicine services for widespread adoption even beyond the pandemic.

## Methods

### Study design

This study used an explanatory mixed methods design consisting of an online survey and in-depth interviews. The qualitative component was guided by grounded theory to study concrete realities of participants and experiences using telemedicine services to render a conceptual understanding of patient satisfaction through an inductive, iterative, and interactive method [21].

### Study participants

Participants were individuals aged 18 to 65 who reside in the Philippines and received telemedicine services during the COVID-19 pandemic.

### Sampling and study size

Convenience sampling was used given the logistical constraints to conduct field data collection during the pandemic. For the online survey, participants were invited through personal and professional networks, telemedicine providers, Facebook, and Instagram. A subsample of the survey participants was invited for an in-depth interview. We selected them based on age, sex, location, and survey answers relating to their telemedicine experience and satisfaction to allow maximum variation sampling, which aims to capture as many population contexts as possible. The chosen respondents were individually contacted through text or email using information they provided in the survey. A total of 200 participants answered the online survey and 16 of them were interviewed.

### Instruments and measures

The online survey questionnaire consisted of items on key socio-demographic characteristics and health-related expenditures, and questions from two validated instruments: 15 questions from Consumer Assessment of Healthcare Providers and Systems (CAHPS) Clinician & Group Adult Visit Survey 4.0 (beta) and 11 questions from Telehealth Usability Questionnaire (TUQ) [22, 23]. CAHPS is a registered trademark of the Agency for Healthcare Research and Quality (AHRQ) with the purpose of advancing our scientific understanding of patient experience with healthcare. TUQ was designed to be a comprehensive questionnaire that covers all usability factors, including usefulness, ease of use, effectiveness, reliability, and satisfaction. The TUQ has acceptable construct validity and internal consistency [24–26]. Telemedicine usability and levels of patient satisfaction were measured for six components (convenience, communication, patient-physician relationship, cost, access, overall satisfaction) using a 5-point Likert scale to rate responses (1: strongly disagree; 2: disagree; 3: neutral; 4: agree; 5: strongly agree), with higher scores reflecting higher usability and satisfaction. Participants were asked how they found out about telemedicine: advertising/paid promotions/endorsements, news, personal research, recommendations by friends or a health professional, social media, or through other means. Participants were also asked on the telemedicine platforms used: SMS (text message), email, video call, voice call, general messaging applications (e.g., Facebook Messenger, Viber), telemedicine-specific platforms (e.g., KonsultaMD, SeeYouDoc, Aide, ClinicKo, Kitika, Medgate, SeriousMD), and others not in the options. Comparisons of the quality of services delivered through telemedicine and in-person were also measured using a 5-point Likert scale of agreement to the following statements: ‘Telemedicine services are the same as in-person consultations” and “Telemedicine services are better than in-person consultations”. A more in-depth response was obtained in the interviews, probing on their telemedicine use and experience, reasons for preferring or not preferring telemedicine over face-to-face, and the factors influencing their telemedicine use and satisfaction. The interview guide was developed and pre-tested following the general themes of the CAHPS and TUQ in English (Additional file 1) and Filipino.

### Data collection procedures

We collected data through an online survey and online interviews from July to November 2021. We used Google Forms for the online survey, while Zoom and Google Meet were used for the interviews. We pre-tested the questionnaire and interview guide in English and Filipino among 15 participants who were similar in characteristics to our study population. The pre-test was conducted online in the same manner as the full-scale survey, and assessed for clarity, organization, and content. The survey and interview guide were revised based on the comments during the pre-testing phase.

All 200 survey participants were asked if they were interested in participating in the interview. To verify that they had used telemedicine services, we included a screening question at the start of the survey to ask about their last telemedicine consultation. For interview participants, we asked them to briefly narrate why and how they used telemedicine. We were unable to confirm through telemedicine providers and managers because of data privacy policies and measures. Among those who consented, we invited participants for an interview through their preferred video call platform (i.e., Zoom, Google Meet). Each interview lasted anywhere from 30 to 120 min. Each interview participant was given approximately USD 3 (USD 1 = PhP 52 as of 11 May 2022) worth of token for participation. Interviews were conducted by AVGN, LMMA, MCCB, DEHD, CBLL, and GATS until data saturation was reached [27]. In our study, we reached data saturation with the 16th participant who no longer provided new data, and where no new emerging themes were observed. Each participant was provided an information sheet and consent form, and time to ask questions prior the start of the interview. The reasons for studying their telemedicine use and satisfaction, and the general background of the research team were also shared with the participants. All participants consented to the interview being recorded.

### Data analysis

#### Quantitative analysis

We analyzed our quantitative data using descriptive statistics: percentage for categorical variables, and median for continuous variables using SPSS Statistics version 25.0 [28]. We described participants according to their age in years, sex (male or female), setting of residence (urban or rural), residence by island group (Luzon, Visayas, Mindanao), educational attainment (secondary or lower, college, post-graduate), employment status (full-time employment, part-time employment, unemployed, student, retired), monthly household income, monthly household health expenditure, monthly individual health expenditure, membership to any health insurance (yes, no), and overall health rating measured as a 5-point Likert scale. Levels of patient satisfaction were measured by computing the frequency and percentage for each item. This analysis is consistent with a study by Ackerman [29] that used TUQ to assess patients’ utilization of and satisfaction with telemental healthcare in the perinatal period. For comparisons between telemedicine services and in-person consultations, we computed the frequency and percentage for both questions with respect to those who answered ‘agree’ and ‘strongly agree’. We classified those who disagreed or strongly disagreed that telemedicine is better than in-person under the theme, “Telemedicine services are inferior to in-person visits”.

#### Qualitative analysis

All interviews were audio-recorded, transcribed verbatim, and translated from Filipino to English. Field notes were made in the course of the qualitative data collection. The researchers are native and fluent speakers of the two languages. Each participant was assigned a unique code to maintain anonymity. The codes or participant IDs presented in this paper are not known to anyone (i.e., providers or patients themselves) outside the research team. Inductive analysis was used to identify emergent themes and patterns from the qualitative data focusing on experiences and satisfaction with telemedicine services, guided by the principles of grounded theory [30]. Transcripts of the interviews were read to identify themes and two research members independently coded the interviews (AVGN, LMMA, MCCB, DEHD, CBLL, GATS) in Microsoft Excel. Each interview was initially coded according to general themes: facilitators or barriers to telemedicine use and satisfaction, which made our preliminary codebook. This codebook was applied to the transcripts and further refined. Once data saturation was reached, we identified emergent themes, which were iteratively reviewed and later finalized (AVGN, LMMA, MCCB, DEHD, CBLL, GATS, AML). Any disagreements were resolved through a consensus. The quotes presented in this paper are either in the original English or translated from Filipino. We report our qualitative findings following the consolidated criteria for reporting qualitative research (COREQ) guideline (Additional file 2).

### Reflexivity

The senior author (AML) has a background in health systems research, health policy, and epidemiology, and has published COVID-19 papers in the Philippine context. The lead and contributing authors (AVGN, LMMA, MCCB, DEHD, CBLL, GATS) have a background in medicine, with males and females well represented in the research team. All authors acknowledge that their perceptions and experiences on health service delivery before and during the pandemic may influence the way data were interpreted. Drawing on the grounded theory approach allowed us to reflect on the richness of experiences by our participants. To ensure we were co-constructing and presenting accurate information, we used coding language that was reflective of the participants’. Finally, we triangulated our qualitative data with findings from the the survey.

## Results

### Participant characteristics

The median age of the 200 individuals who participated in our survey was 31.50 years (IQR 23.5–46.0 years). More than half of our study population were female (64.0%) and college graduates (65.0%). A total of 62 participants worked full-time (31.0%) and 162 resided in Luzon (81.0%). The median monthly household income of participants was USD 577, which is considered part of the lower middle-income class in the Philippines [31]. The median monthly household health expenditure was USD 69 and the median monthly individual health expenditure was USD 38. Only half availed of any health insurance (51.5%). The median overall health rating of participants was 4 out of 5 (Table 1).

**Table 1:** Characteristics of survey respondents (*n* = 200)

Characteristic	N (%)^a^
Median age in years (IQR)	31.50 (23.5–46.0)
Sex	
Female	128 (64.0)
Male	72 (36.0)
Location by urbanicity	
Urban	170 (85.0)
Rural	30 (15.0)
Residence by island group	
Luzon	162 (81.0)
Visayas	28 (14.0)
Mindanao	10 (5.0)
Highest educational attainment	
Secondary or lower	35 (17.5)
College	130 (65.0)
Post-graduate	35 (17.5)
Employment status	
Full-time employment	62 (31.0)
Part-time employment	15 (7.5)
Unemployed	36 (18.0)
Student	48 (24.0)
Retired	8 (4.0)
Median monthly household income in USD (IQR)	577 (231–1,923)
Median monthly household health expenditure in USD (IQR)	69 (38–115)
Median monthly individual health expenditure in USD (IQR)	38 (19–77)
Median overall health rating (IQR)	4 (3–4)
Availed any health insurance	
Yes	103 (51.5)
No	97 (48.5)

^a^May not total to 100% because of missing data

The median age in years of the 16 interview participants was 29. More than half (62.5%) were female. Thirteen (81.25%) participants were from Luzon, two (12.5%) were from Visayas, and one (6.25%) was from Mindanao. More than half (68.75%) of the participants availed of health insurance. Only seven (43.75%) participants disclosed their monthly household income with a median of USD 577.

### Overall telemedicine usability and satisfaction

**Fig. 1:**
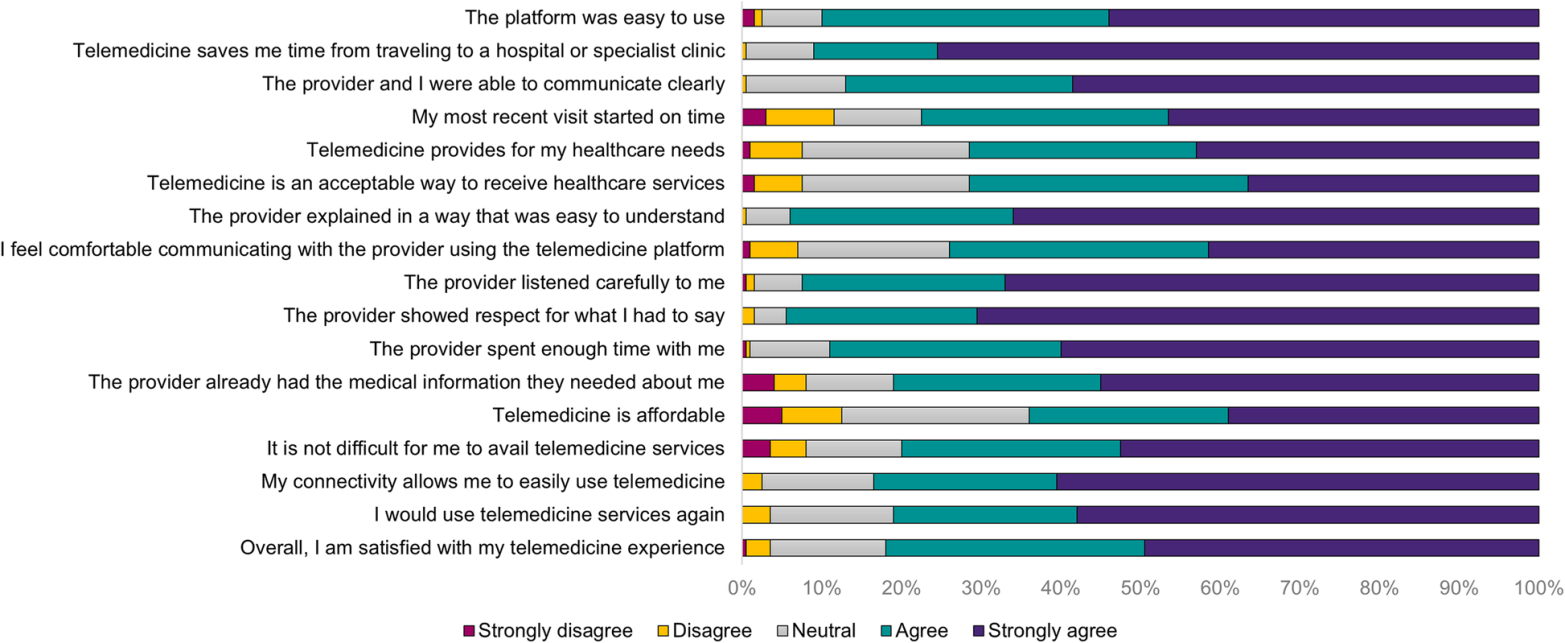
Telehealth Usability Questionnaire (TUQ) ratings for telemedicine services in the Philippines during the COVID-19 pandemic (*n* = 200)

Across all statements, most participants agreed that they were satisfied with telemedicine services in terms of convenience, communication, patient-physician relationship, cost, and access. Of these reasons, convenience was identified by majority of the participants (91.0%) to positively influence their telemedicine use and satisfaction, saving them time from traveling to a hospital or specialist clinic. On the other hand, 3 in 5 perceived telemedicine to be affordable. A number of participants provided lower ratings to communication, patient-physician relationship, and access items in the TUQ. Only 116 survey participants (58.0%) strongly agreed that they would use telemedicine services again (Fig. 1).

### Comparisons between telemedicine and in-person consultations

#### Telemedicine services are the same as in-person consultations

A total of 68 survey participants agreed (34.0%) that the service provided through telemedicine was the same as in-person consultations. This is supported by our qualitative findings that just like face-to-face consultations, telemedicine allows patients to access services provided by physicians, express their medical concerns, and have their concerns addressed. This perception of adequacy of care provided via telemedicine promotes its use:

“[In a way,] telemedicine is the same [as face-to-face consultation] because I still get to talk to a doctor. You get to voice out your problems or your medical history, and then get a prescription or diagnosis.” (E131, 20–24 years old, female)

#### Telemedicine services are better than in-person consultations

A total of 72 survey participants (36.0%) perceived telemedicine services to be better than in-person consultations. One participant who used KonsultaMD for a skin condition mentioned convenience and experiencing better quality of service:

“[Telemedicine is] just so much more efficient and convenient, and I feel like the doctors are not in a rush to get to the next patient, and they really try to [serve] you better over telehealth as compared to face-to-face consultations.” (E3, 20–24 years old, female)

#### Telemedicine services are inferior to in-person consultations

A total of 60 survey participants (30.0%) perceived telemedicine services to be inferior to in-person consultations. Interview participants elaborated and expressed that telemedicine is lacking in multiple functions of care including laboratory tests and diagnostics, physical assessment, and rapport-building. Their preference for telemedicine or in-person visit depended on the health condition. A participant with an atypical presentation of her illness who needed multiple laboratory tests for her diagnosis: “I would never use telehealth consultation again for other matters besides follow-up care.” (E69, 20–24 years old, female). She explained that the telemedicine consultation was not useful because she still needed to do an in-person consultation to have her concerns addressed. She also mentioned that whether or not she did telemedicine or an in-person consultation, she still had to be at the hospital for laboratory results.

Several respondents noted that some diseases cannot be assessed through telemedicine due to the necessity for certain equipment or physical assessment, leading to the preference for face-to-face consultations:

“For those illnesses that cannot be diagnosed by video call, like those that need additional equipment to check, then it’s better to do it face-to-face.” (E133, 25–29 years old, female)

“I used to have skin asthma. So for me, it’s really necessary to go and see a dermatologist so that he/she can physically see what rashes I have.” (E93, 40–44 years old, female)

## Factors influencing telemedicine use and satisfaction

### Facilitators

#### Safety of telemedicine during the pandemic

All 16 interviewees cited COVID-19-related reasons for their telemedicine consultations. Many participants used telemedicine because of the possibility of being exposed to the virus on the way or at the place of face-to-face consultation itself:

“Telemedicine has less exposure [to the coronavirus], less travel time and it’s also related to my mental health wherein I really don’t want to leave the house.” (E128, 25–29 years old, female)

“With the pandemic, of course you’d choose to not expose yourself further. If you’re already sick, you don’t want to expose yourself to an additional kind of virus that’s more deadly.” (E133, 25–29 years old, female)

#### Telemedicine offers options that maintain privacy

A number of interviewees preferred telemedicine because privacy could be maintained. Being able to be discuss their chief complaints and answer questions in history taking pertaining to their private areas were some of the reasons they chose telemedicine:

“Telemedicine is convenient for me. You’re still one-on-one with the doctor. For example, either I’m in the living room or in my bedroom. Pre-pandemic – in the clinic, sometimes there are other doctors who share a cubicle. Especially when the doctor is asking questions regarding your private area, sometimes you are ashamed to mention it because others might hear.” (E97, 35–39 years old, female).

“In my case, I couldn’t go out [because] it’s a sexual concern.” (E131, 20–24 years old, female).

#### Telemedicine is affordable due to overall reduced costs

Around 128 survey participants (64.0%) identified low cost of telemedicine services as a facilitator. Interviewees supported this and mentioned being able to save on transportation costs and doctor’s fees:

“For me, I was able to save with telemedicine. I’m not sure if it depends on the doctor’s fee, but so far it seems that the doctor’s fee is cheap and only costs around USD 7–11 per consultation.” (E97, 35–39 years old, female)

#### Telemedicine is convenient due to reduced time and travel requirements

Most of the survey participants (91% ) identified convenience as a facilitator to telemedicine use and satisfaction for the following reasons: decrease in waiting time which additionally removes the necessity to file a leave of absence to go to the doctor, the absence of traffic, and the elimination of niceties when going outside such as taking a bath and dressing up:

You don’t have to get dressed, and drive or take a Grab.” (E131, 20–24 years old, female)

“There are more chances that the video chat will definitely save a lot more time. There’s no travel time, there’s no waiting time.” (E79, 25–29 years old, male)

“With telemedicine – generally at least in my experience, the waiting time is reduced so I think in that regard it’s nice.” (E74, 20–24 years old, female)

#### Telemedicine is easily accessible and readily available

Another facilitator to telemedicine use and satisfaction was accessibility of services, which was identified by 160 survey participants (80.0%). The interviewees explained that telemedicine services were available at any time and did not require them to see their doctor physically:

“Access [was one of the reasons why I chose to use telemedicine] because you just wait in the house and/or the doctor’s availability.” (E56, 50–54 years old, male)

“I like telemedicine because when you need it and you’re far from your doctor, you can just call, and describe and maybe send pictures or information. You can still get your medicine and advice from the doctor.” (E22, 40–44 years old, female)

#### Telemedicine offers more avenues of communication

Telemedicine offers more avenues for communication as its scope includes text-based messaging, voice calls, and video calls across different platforms. In the interviews, several platforms were identified including KonsultaMD, SeriousMD, and Aide. Messenger and Viber were noted by some participants to be convenient applications for communication because they can reach out to their provider anytime. Hospital hotlines and school medical services were also platforms mentioned by interviewees. This was perceived as a benefit of telemedicine in itself:

“If you are out of the WiFi zone, it is hard to connect with video call. You have the option to email, text, or call.” (E11, 55–59 years old, female).

Survey respondents identified the use of one or more of the following: SMS, general messaging applications, email, video call, voice call, telemedicine-specific platforms, and others (i.e., dedicated hotlines). About half have used messaging applications (50.0%) and video call (45.5%) for telemedicine. Email (11.5%) is least used for telemedicine (Table 2).

**Table 2:** Applications and platforms of telemedicine (*n *= 200)

Telemedicine applications and platforms	N (%)^a^
SMS	54 (27.0)
General messaging applications (Messenger, Viber)	100 (50.0)
Email	23 (11.5)
Video call	91 (45.5)
Voice call	63 (31.5)
Telemedicine-specific platforms (e.g., SeriousMD, KonsultaMD)	46 (23.0)
Others (e.g., hospital hotlines)	3 (1.5)

^a^N does not total to 200 since each participant was permitted to select all methods of telemedicine they have used

### Barriers

#### Limited to absent prior patient-physician relationship

Of the 200 survey participants, 162 or 81.0% had an existing medical record with their telemedicine provider. For some, this limited to no previous physician-patient relationship contributed to dissatisfaction with telemedicine services:

“That’s also the weakness of that telemedicine platform [redacted]. It’s because you’re queueing for doctors, for GP [general practitioner] doctors, right? What happens is that you don’t get to choose. Whoever is available, that’s who your provider will be.” (E69, 20–24 years old, female)

#### Perceived lack of experience and ability to provide quality service among telemedicine providers

Related to the absence or lack of prior-physician relationship, depending on the telemedicine service and platform used, some interview participants were unable to choose a physician whom they preferred and perceived to be able to meet their needs:

“For emergency cases, it’s mostly resident doctors who would answer [the telemedicine hotline], not really a doctor [consultant/attending]. I experienced that in [redacted hospital 1], they weren’t sure if they should ask their superior, or rather the department head of dermatology, how my case should be managed. This means they couldn’t make decisions on the spot about what should be done to the patient, unlike in [redacted hospital 2], decision making is automatic because it’s really a doctor [consultant/attending] answering.” (E15, 30–34 years old, male)

#### Inherent limitations of telemedicine

Almost 3 in 10 of our survey participants disagreed, or were neutral, on telemedicine being an acceptable way to receive healthcare services. Interviewees expressed concerns on quality of services provided through telemedicine due to its limitations, especially for conditions that require diagnostic tests and physical check-ups. Doctors ask several questions and seek validation from patients. There is also perceived poorer quality of care because patients feel that they are not being checked thoroughly by the doctor:

“I don’t think the consultation can provide enough accuracy compared to an in-person consultation for the prescribing doctor. I don’t think an over-the-phone conversation can truly give a telemedicine provider an accurate evaluation of myself.” (E89, 25–29 years old, male)

“It’s really different when the doctor looks at you, puts his stethoscope on you, and conducts physical assessment. Unlike in the past, the doctors will immediately check the part of your body that is painful.” (E71, 50–54 years old, male)

#### Perceived high costs that are comparable to in-person consultations

The cost of telemedicine was perceived as a barrier to the use and satisfaction of telemedicine services with some participants expecting that telemedicine costs would be lower. As one interviewee remarked: “I really expected it [telemedicine] to be cheaper than an in-person consultation. If I’m going to pay the same price, then I’ll choose to go to a health facility.” (E82, 15–19 years old, male).

In the survey, the cost of telemedicine ranged from USD 0 to USD 192 with a median of USD 7.5. Telemedicine consultations for mental health conditions were in the higher range. Nearly half (42.5%) of the survey participants had consultations for free with some relying on promotions to avail telemedicine. There were also participants who perceived the price of telemedicine to be expensive and inaccessible for other population groups: “I was just thinking in general, how would Filipinos –from all demographics, all social classes – how would they find it? So I said it [the cost] might be a barrier for some.” (E131, 20–24 years old, female).

#### Poor network connectivity resulting to ineffective communication

Ineffective communication as a result of poor network connectivity was identified as a barrier by five (2.5%) survey participants. One interview participant noted: “Even if you’re connected and you’re talking, sometimes the telemedicine provider doesn’t hear what you’re saying, or vice versa. They hear you, but they don’t understand because of poor call quality [due to poor connectivity].” (E84, 50–54 years old, male). Of the 200 survey participants, one disagreed and 25 were neutral when asked if they were able to communicate with their provider clearly. Others also mentioned that their satisfaction with telemedicine depends on how smoothly the telemedicine consultation goes, which in turn is significantly influenced by internet connectivity, the platform’s data usage, and the gadgets used for the consultation: “If we’re in the middle of a serious discussion, then suddenly it [the internet connection] will be cut off? It’s awkward and embarrassing, especially if I don’t know the doctor.” (E93, 40–44 years old, female).

#### Inaccessibility of required technology interferes with telemedicine use and satisfaction

Participants identified access to technology required for the consultation to be an important consideration, and cited inaccessibility as a contributing factor to its disuse and dissatisfaction: “People don’t have mobile load. Some don’t have good cameras for their phones or gadgets, or some don’t have it. I think that’s the disadvantage of using telemedicine.“ (E21, 40–44 years old, female).

## Discussion

Our study showed that Filipino patients are generally satisfied with the services provided through telemedicine applications and platforms. This is consistent with previous studies that report high levels of patient satisfaction [32–34]. Telemedicine was perceived to be similar to in-person consultations in that the participants were able to obtain medical advice and have their health concerns addressed regardless of the mode of delivery. Some perceived it to be better primarily because of convenience and accessibility. However, the inherent limitations of telemedicine restrict its utility, especially for health conditions that require physical assessments and laboratory tests.

We found that telemedicine use and satisfaction are positively influenced by a number of factors including safety during the pandemic, privacy, affordability, convenience and accessibility, and availability of more avenues of communication. Safety was a major concern that prompted participants to use telemedicine. Telemedicine enables patients to avoid situations that would expose them to SARS-CoV-2, the causative agent of COVID-19, such as traveling and staying for long periods in high-risk environments. These safety concerns, together with lockdown restrictions, resulted to significant declines in hospital admissions for non-urgent procedures [10]. As a result, digital health solutions and telemedicine have been introduced to respond to health concerns while reducing the risks to the patient and the burden to the health system [34]. Participants also mentioned that telemedicine allowed them to maintain privacy, which is not always possible for in-person consultations. The benefits of anonymity are especially important with regards to sensitive and potentially stigmatizing health issues such as mental or sexual health conditions [29]. Because telemedicine removes the need to travel, participants also viewed it as more affordable, accessible, and convenient. This was noted by participants as an enabling factor to use telemedicine, especially since a third of our participants are full-time employees and a quarter are students. Traveling for healthcare purposes could mean missing work or school [35], and telemedicine therefore gives them greater ability to manage their time around consultations. Additionally, the variety of communication modes and platforms available contributed to patient use and satisfaction [36]. This enables patients and providers to communicate through other avenues when technical difficulties arise.

Barriers to telemedicine use and satisfaction included: perceived poor quality of care and service due to limited or absent prior physician-patient relationship and inability to choose preferred providers, inherent limitations of telemedicine, perceived high costs that are comparable to in-person consultations, and poor internet connectivity and other technological barriers (e.g., gadget availability and specification). Lack of trust in the physician can leave the patient dissatisfied with the service provided, which can affect adherence to doctor’s advice or prescribed treatment plan [37]. Established relationships are an important factor in telemedicine use, as patients are less willing to use telemedicine to see a provider that they do not know [38, 39]. Telemedicine was perceived as an efficient and effective alternative to in-person consultations, but not for all health conditions especially those requiring physical assessments and diagnostics. Other studies have reported similar findings [40–42], with providers having to determine when a telemedicine is most useful vis-à-vis a face-to-face visit, considering their risks and benefits, and the needs of the patient. Innovative solutions such as automated logic flows (bots) for referrals and scheduling, tools such as video otoscopes and electronic stethoscopes for examination, and artificial intelligence technologies can be explored [1]. Studies comparing these modes of healthcare delivery and traditional face-to-face method need to be carried out to ensure effective, accurate, and quality care is provided. While some participants in our study and in other published literature noted cost as a factor contributing to patient satisfaction [5, 34, 43], we also found it to be a reason for dissatisfaction among our participants. This may be due to the significant proportion of participants in our sample who were not employed with almost a half not enrolled in any health insurance plan. The Implementing Rules and Regulations (IRR) of the Universal Health Care (UHC) Act stipulates that the Philippine Health Insurance Corporation or PhilHealth shall use its contracts to incentivize the integration and use of telemedicine [18]. The PhilHealth “*Konsultasyong Sulit at Tama*” (or *Konsulta*) package is a comprehensive outpatient benefit that integrates telemedicine to ensure access to services [44]. According to a PhilHealth circular released in 2021 [45], home isolation services including telemedicine will be incentivized as long as Konsulta providers have accomplished all necessary documents. In addition, several health maintenance organizations in the Philippines reimburse telemedicine consultations or provide these services for free among insured patients, which lessens financial burden [46–48]. While medical insurance and benefit packages allow access and utilization of healthcare services at lower costs [49], the rollout of the Konsulta package has been significantly delayed and therefore, patients need to pay out-of-pocket to avail of services including telemedicine. Some of our participants availed promotions to get free telemedicine consultations, however this may not be sustainable for both provider and patient. Poor network connectivity and technological barriers resulting to ineffective communication also decreased levels of patient satisfaction, which is consistent with published findings [50]. These barriers are especially significant in the Philippines, where service delivery and resources are inequitably distributed and affected by the pandemic [9, 19] – further compounded by the limited access to the Internet and technology in remote areas. As a result, telemedicine is not widely adopted in these communities. These technological issues and other barriers identified by the study need to be addressed to provide services to patients where physicians are few, and where long-distance care is most needed [35].

### Limitations

A number of limitations need to be considered when interpreting our findings. First, the results of the study are influenced by the social context and implications of the COVID-19 pandemic during the time the study was conducted. Because of this, scores provided by the participants are not indicative of telemedicine alone, but rather, indicative of patient satisfaction when using telemedicine in the context of the COVID-19 pandemic. Patient telemedicine satisfaction studies also generally report high ratings reflective of their experiences with healthcare and service delivery [50]. However, we addressed this issue by including a measure on preference between telemedicine and face-to-face consultation [50]. A considerable proportion of participants reported the same level of satisfaction for both modes of service delivery, while a few interview participants reported less satisfaction for telemedicine. This confirms in part that telemedicine satisfaction is high in our study because of their experience with telemedicine itself, and not only because of the general care they receive from the health system. Second, our use of convenience sampling for the descriptive quantitative study and online data collection methods potentially excluded participants from low-resource and remote communities who may have other experiences and where the impact of barriers may be more profound. This limits the generalizability of our findings to those who share similar characteristics with the study participants. While we attempted to interview participants with different backgrounds and experiences to address this issue, majority of those who were willing and consented were still mainly from urban areas. However, we were still able to capture issues of cost and technology, which are also barriers to use and satisfaction among individuals from rural and lower-resource communities. Third, we asked their general experience and satisfaction to telemedicine regardless of platforms used. We are therefore unable to disentangle the effect of specific telemedicine platforms on satisfaction and use. Despite these limitations, our study provides a rich source of data, contributing to the evidence that telemedicine can be integrated into routine care during and beyond the pandemic while offering insights into use and satisfaction through the lens of patients in a low-and-middle income country.

## Conclusion

This study showed that Filipino patients are generally satisfied with telemedicine services provided during the COVID-19 pandemic. Telemedicine use and satisfaction are influenced by factors related to the individual (e.g., cost, perceived safety, convenience), health provider and system (e.g., perceived competence of providers, physician-patient relationship), and external factors such as connectivity and technological demands. Our findings also suggest that participants have varying reasons for perceiving telemedicine to be equal, inferior, or better than in-person consultations. Telemedicine was viewed as safe, efficient, and effective especially when barriers are removed, but only for health conditions that do not require physical examination and laboratory tests. Expectations of patients on the costs, as well as the conditions that can be addressed through telemedicine, need to be managed and discussed by providers to increase satisfaction. Continued adoption of telemedicine will require improvements in technology and better patient communication to meet their needs. Our study points to the following recommendations: (a) Integration of telemedicine services in geographically remote areas that lack access to medical services to advance the UHC agenda of providing quality healthcare to all; (b) Strengthening of infrastructure to allow the use of devices and Internet for telemedicine with little to no interruption; (c) Training and performance evaluation of telehealth providers to ensure quality telemedicine services; (d) Patient communication on telemedicine and its limitations; (e) Patient support for those with technological difficulties; and (f) Future research to include stakeholder perspectives and patient experiences from remote and low-resource communities.

## Supplementary Information

**Additional file 1.** Interview guide (English).

**Additional file 2.** COREQ reporting guideline.

## Data Availability

The quantitative dataset generated and/or analyzed during the current study is available from the corresponding author on reasonable request. The dataset generated during individual interviews are not publicly available due to the information provided to the participants when obtaining their informed consent, stating that all attempts would be made to maintain confidentiality.
