# Supplementary material for: Patient satisfaction with telemedicine in the Philippines during the COVID-19 pandemic: a mixed methods study
Source: BMC Health Serv Res. 2023 Mar 22;23:277. doi: 10.1186/s12913-023-09127-x (PMC10032251; doi:10.1186/s12913-023-09127-x)
Supplement: Supplementary file 1 — Additional file 1. Interview guide (English). [file 12913_2023_9127_MOESM1_ESM.docx]

**Additional file 1**. Interview guide (English)

**A. Overview**

A1 How did the platform(s) you used affect your telemedicine experience?

A2 What was the process for conducting telemedicine in your chosen platform?

A3 How does telemedicine affect your health-seeking behavior?

A4 Would you use telemedicine again? Why or why not?

**B. Telemedicine vs. Face-to-Face Consultations**

B1 What are your reasons for using telemedicine instead of going for a physical consultation?

B2 What do you think about telemedicine as an alternative to face-to-face consultation?

B3 Was the transition from in-person consultations to telemedicine easy or difficult? What were the factors that made the transition easy or difficult?

B4 What is the biggest difference between telemedicine and in-person consultation for you?

B5 In what scenarios would you prefer telemedicine?

B6 In what scenarios would you prefer a physical consultation?

B7 Can telemedicine replace the physical consultation for your primary care needs? Why or why not?

**C. Effect of COVID-19 pandemic on telemedicine**

C1 How does/did the COVID-19 pandemic affect your health-seeking behavior especially with regards to telemedicine?

C2 Do you think the Philippines is ready to embrace telemedicine during and beyond the pandemic?

**D. Participant-specific questions based on answers to the survey**

D1 Your survey answers also show that you had [number] telemedicine consultations. Was your overall experience satisfactory? (Ask them to compare their experience with face-to-face consultations before the pandemic)

D2 Would you say that the cost of telemedicine might eventually be too expensive for you in the long run?

(If yes) What would your alternative be in case telemedicine becomes too costly for you?

(If no) When the pandemic is over, would you still prefer to use telemedicine over face-to-face check-ups?

D3 With the challenges you have mentioned, are your telemedicine consultations becoming progressively better or worse?

(If worse because of the Internet) Do you think a third telecommunications company will improve your connectivity problems?

D4 Your survey mentions that your most recent telemedicine consultation was about your [health condition]. Did you find relief after your consultation? (Yes or no. Explain)

Have you had face-to-face consultations about your [health condition] before the pandemic?

(If yes) How often?

Compare your telemedicine and your face-to-face consultations. Which one would you prefer?

D5 Are the platforms you used easily understandable or complicated?

(If complicated) How did you access it?

D6 Additional questions and/or other insights from the participant
